# Pleiotropic Associations of Allelic Variants in a 2q22 Region with Risks of Major Human Diseases and Mortality

**DOI:** 10.1371/journal.pgen.1006314

**Published:** 2016-11-10

**Authors:** Alexander M. Kulminski, Liang He, Irina Culminskaya, Yury Loika, Yelena Kernogitski, Konstantin G. Arbeev, Elena Loiko, Liubov Arbeeva, Olivia Bagley, Matt Duan, Arseniy Yashkin, Fang Fang, Mikhail Kovtun, Svetlana V. Ukraintseva, Deqing Wu, Anatoliy I. Yashin

**Affiliations:** Biodemography of Aging Research Unit, Social Science Research Institute, Duke University, Durham, NC United States of America; Stanford University School of Medicine, UNITED STATES

## Abstract

Gaining insights into genetic predisposition to age-related diseases and lifespan is a challenging task complicated by the elusive role of evolution in these phenotypes. To gain more insights, we combined methods of genome-wide and candidate-gene studies. Genome-wide scan in the Atherosclerosis Risk in Communities (ARIC) Study (N = 9,573) was used to pre-select promising loci. Candidate-gene methods were used to comprehensively analyze associations of novel uncommon variants in Caucasians (minor allele frequency~2.5%) located in band 2q22.3 with risks of coronary heart disease (CHD), heart failure (HF), stroke, diabetes, cancer, neurodegenerative diseases (ND), and mortality in the ARIC study, the Framingham Heart Study (N = 4,434), and the Health and Retirement Study (N = 9,676). We leveraged the analyses of pleiotropy, age-related heterogeneity, and causal inferences. Meta-analysis of the results from these comprehensive analyses shows that the minor allele increases risks of death by about 50% (*p* = 4.6×10^−9^), CHD by 35% (*p* = 8.9×10^−6^), HF by 55% (*p* = 9.7×10^−5^), stroke by 25% (*p* = 4.0×10^−2^), and ND by 100% (*p* = 1.3×10^−3^). This allele also significantly influences each of two diseases, diabetes and cancer, in antagonistic fashion in different populations. Combined significance of the pleiotropic effects was *p* = 6.6×10^−21^. Causal mediation analyses show that endophenotypes explained only small fractions of these effects. This locus harbors an evolutionary conserved gene-desert region with non-coding intergenic sequences likely involved in regulation of protein-coding flanking genes *ZEB2* and *ACVR2A*. This region is intensively studied for mutations causing severe developmental/genetic disorders. Our analyses indicate a promising target region for interventions aimed to reduce risks of many major human diseases and mortality.

## Author Summary

Biomedical research and medical care are traditionally focused on individual health conditions in order to postpone, ameliorate, or prevent the accumulation of morbidities in late life. An attractive idea is to find factors, which could reduce burden of not just one disease but a major subset of them to efficiently extend healthy lifespan. Here we focus on the analyses of genetic predisposition to risks of major human age-related diseases and mortality. The analyses highlight a locus in band 2q22.3 associated with risks of coronary heart disease, heart failure, stroke, diabetes, cancer, neurodegenerative diseases, and death. Our analyses indicate a promising target region for interventions aimed to reduce risks of many major human diseases and mortality.

## Introduction

The demographic transition on aging of populations in developed countries requires strategies, which could extend healthspan and lifespan, and compress morbidity [1–3]. Breakthroughs in genome-wide sequencing and high-throughput genotyping raised enthusiasm for advancing the progress in the field by discovering genes influencing various health-related traits. To accelerate the progress, one necessarily faces the need to deal with genetic predisposition to complex, inherently heterogeneous, age-related traits, i.e., traits that are characteristic of the elderly people in modern societies.

Heterogeneity is the result of various processes. Of the most familiar sources of heterogeneity, genome-wide association studies (GWAS) commonly handle those associated with evolutionarily selected genetic patterns in populations [4] and complex etiologies of human health-related phenotypes [5].

Age-related traits are a special case of heterogeneous phenotypes, because they emerge as a new *widespread* phenomenon in humans [6], especially given substantially shorter lifespan of our even recent predecessors [7], and because they are characteristic of the post-reproductive period, when the role of evolutionary selection in these traits is elusive [6,8–12]. These factors imply that “diseases are not shaped by selection,” [6], i.e., evolution did not fix the molecular basis of age-related traits. The latter makes the analyses of genetic influence on such traits be a more challenging task than that of fitness-related traits (e.g. height [13]). An important challenge is a special type of heterogeneity attributed to the elusive role of evolution in shaping the genetic basis of age-related traits. This heterogeneity is the result of age-related processes in an organism and compositional changes in a population in changing environment [9,14,15].

The age-related heterogeneity implies the potential existence of gene-endophenotype-phenotype pathways (i.e., mechanisms mediating the effects between genes and age-related traits) and that these mechanisms can change with age, time, and population composition even if the same genetic variant and trait are considered. This heterogeneity can naturally contribute to non-replication of genetic effects in different populations even in case of populations of the same ancestry and phenotypes [16–18].

The elusive role of evolution in fixing molecular basis of age-related traits can also benefit genetic analyses because it can enhance the basis of pleiotropic influences on different traits, including “apparently distinct” ones [19]. Statistical benefit is that pleiotropic analysis may improve power [20]. Substantive benefit is that pleiotropic influences on apparently distinct traits are a part of an attractive gerontological idea which has been conceptualized as geroscience [21]. This concept assumes that age [22] and aging [23] can be major risk factors of geriatric diseases of distinct etiologies [24]. Detecting such pleiotropy and developing gene-based interventions may strengthen strategies for reducing burden of not just one disease but a major subset of them to efficiently extend healthspan and lifespan [23,25,26].

Given specific properties of age-related traits, common methods of GWAS of these traits may be insufficient and more comprehensive methods typical for candidate gene studies may be needed. One strategy to improve genetic analyses of age-related traits is to use genome-wide scan for pre-selection of promising SNPs and more comprehensive methods for detail analyses of these SNPs in large samples.

Following this approach, we selected promising SNPs from GWAS of the Atherosclerosis Risk in Communities (ARIC) Study (see Methods). Then we conducted detail analysis of associations of SNPs at a promising locus in band 2q22.3 with risks of major diseases including coronary heart disease (CHD), heart failure (HF), stroke, diabetes, cancer, and neurodegenerative diseases (ND, dementias including Alzheimer’s type) and risks of death using the ARIC, the Framingham Heart Study (FHS), and the Health and Retirement Study (HRS). We leveraged the analyses of pleiotropy, age-related heterogeneity, and causal inferences. In causal mediation analyses, we used biomarkers (body mass index [BMI], total [TC] and high-density lipoprotein [HDL-C] cholesterols; triglycerides [TG], systolic [SBP] and diastolic [DBP] blood pressures) as endophenotypes for diseases or death and diseases as endophenotypes for death. We show that the minor allele increases risks of death by about 50% (*p* = 4.6×10^−9^), CHD by 35% (*p* = 8.9×10^−6^), HF by 55% (*p* = 9.7×10^−5^), stroke by 25% (*p* = 4.0×10^−2^), and ND by 100% (*p* = 1.3×10^−3^). This allele also significantly influences each of two diseases, diabetes and cancer, in antagonistic fashion in different populations. Combined significance of the pleiotropic effects was *p* = 6.6×10^−21^.

## Results

Basic characteristics of the ARIC, FHS, and HRS genotyped participants relevant to our analyses and the available sample sizes for carriers and non-carriers of the minor allele (rs222826_T in ARIC and FHS and rs222827_A in HRS) are given in Table 1 (see Methods).

**Table 1:** Basic characteristics of carriers and non-carriers of the minor allele of rs222826 or rs222827 SNPs in the selected studies.

Factors	ARIC, rs222826		FHS_C1, rs222826		FHSO, rs222826		HRS, rs222827	
	CC	CT+TT	CC	CT+TT	CC	CT+TT	GG	GA+AA
N (%^a^)	9154 (95.6)	419 (4.4)	982 (94.7)	55 (5.3)	3184 (93.7)	213 (6.3)	9308 (96.2)	368 (3.8)
Age, mean (SD), years	54.3 (5.7)	54.6 (5.6)	37.9 (6)	37.3 (5.9)	35.4 (10)	35.1 (9.7)	58.1 (9.1)	58 (9)
LS, mean (SD), years	69.8 (5.9)	69.8 (5.8)	88.2 (7.1)	87.7 (6.5)	72.1 (9.2)	70.9 (9.1)	75 (10.2)	74.7 (10.1)
BC, mean (SD), years	1933.3 (5.8)	1933.1 (5.7)	1909.7 (6.1)	1910.1 (5.7)	1935.1 (9.9)	1935.5 (9.4)	1940.2 (10.3)	1938.5 (10.3)
BMI, mean (SD), kg/m^2^	27 (4.8)	27.4 (5.1)	24.7 (3.7)	23.5 (3.2)	25.2 (4.3)	25.7 (4.3)	26.9 (5)	27 (5.2)
TC^c^, mean (SD), mg/dl	214.9 (40.8)	217 (40.3)	238.3 (44.2)	239.3 (42.4)	194.3 (38)	194.5 (39)	194.6 (44.3)	192.1 (44.2)
HDL-C^c^, mean (SD), mg/dl	50.6 (16.7)	48.6 (16.1)	49.6 (14.5)	49.4 (16.1)	50.9 (14.6)	50.1 (14.6)	56.1 (15.7)	55 (16.4)
TG^c^, mean (SD), mg/dl	136.9 (91.4)	149.4 (104.9)	151.2 (99.9)	151 (86.8)	91.9 (85.5)	92.9 (71.1)	NA	NA
SBP, mean (SD), mmHg	139 (15.5)	139.2 (16.7)	126.2 (15.1)	123 (11.2)	120.2 (14.5)	121.3 (14.7)	NA	NA
DBP, mean (SD), mmHg	92 (9.8)	92.2 (9.4)	79.5 (9.6)	78.7 (8.1)	77.7 (10)	78.3 (10.3)	NA	NA
CHD, yes (%^b^)	846 (9.2)	62 (14.8)	385 (39.2)	23 (41.8)	557 (17.5)	36 (16.9)	3579 (38.5)	149 (40.5)
HF, yes (%^b^)	766 (8.9)	49 (12.6)	272 (27.7)	18 (32.7)	237 (7.4)	21 (9.9)	NA	NA
Stroke, yes (%^b^)	507 (5.5)	28 (6.7)	191 (19.5)	16 (29.1)	191 (6)	17 (8)	1184 (12.7)	46 (12.5)
Diabetes, yes (%^b^)	1608 (18)	94 (22.9)	305 (31.1)	13 (23.6)	312 (9.9)	29 (13.7)	2375 (25.5)	116 (31.5)
Cancer, yes (%^b^)	1225 (13.4)	66 (15.8)	467 (47.6)	28 (50.9)	1117 (35.1)	81 (38)	2213 (23.8)	71 (19.3)
ND, yes (%^b^)	NA	NA	266 (27.7)	24 (43.6)	NA	NA	NA	NA
Death, yes (%^b^)	1344 (14.7)	79 (18.9)	880 (89.6)	54 (98.2)	656 (20.6)	60 (28.2)	1230 (13.2)	59 (16)

HRS SNP used is proxy SNP rs222827, which is in perfect linkage disequilibrium (LD), *r*^*2*^ = 1, with rs222826 in CEU population; these SNPs are 90 bp apart.

Percentage is within

^a^ the genotyped sample

^b^ each genotype.

N denotes sample size; NA = not available.

Mean age is given at baselines; LS denotes lifespan measured by age at death or the end of follow-up; BC denotes birth cohorts.

ARIC = the Atherosclerosis Risk in Communities Study, FHS_C1 = the Framingham Heart Study (FHS) original cohort; FHSO = the FHS Offspring cohort, and HRS = the Health and Retirement Study.

BMI = body mass index; TC = total cholesterol; HDL-C = high-density lipoprotein cholesterol; TG = triglycerides; SBP = systolic blood pressure; DBP = diastolic blood pressure; CHD = coronary heart disease; HF = heart failure; and ND = neurodegenerative diseases (dementias including Alzheimer’s type).

^c^ Means of TC, HDL-C, and TG concentrations are given at 9^th^ FHS_C1 examination because this visit included the largest sample size with non-missing information on these lipids. For FHSO and other studies means of lipid concentrations were representatively given at baselines.

### Risks of diseases and death

Table 2 shows the estimates of risks of major human diseases or death for carriers and non-carriers of the minor allele. This allele is highly significantly associated with risks of CHD in ARIC (HR = 1.74, *p* = 1.1×10^−7^) and death in FHS (HR = 1.64, *p* = 1.3×10^−6^). Leveraging these pleiotropic associations, the global null hypothesis that neither of these associations is true evaluated using the Fisher’s combined probability test [27] is *p* = 4.7×10^−12^. This result implies that the probability of being a false finding in this case is much smaller than that defined by the genome wide significance (*p*_*GW*_ = 5×10^−8^). Multiple testing correction for 15 tests with other phenotypes, which are not included in the Fisher’s test (see Table 2), does not alter this result, *p* = 4.7×10^−12^ × 15 = 7.1×10^−11^ << *p*_*GW*_.

**Table 2:** Hazard ratios for major human diseases and death for the rs222826 ^a^ minor allele carriers in ARIC, FHS, and HRS.

Outcome	ARIC, N = 9,618			FHS, N = 4,700			HRS^a^, N = 9,735		
	HR	95% CI	p-value	HR	95% CI	p-value	HR^b^	95% CI	p-value
CHD	1.74	1.42–2.14	1.1E-7	1.07	0.82–1.41	6.1E-1	1.10	0.88–1.38	3.8E-1
HF	1.50	1.12–2.01	6.1E-3	1.52	1.09–2.12	1.5E-2	NA	NA	NA
Stroke	1.18	0.75–1.85	4.7E-1	1.70	1.19–2.43	3.6E-3	1.01	0.72–1.38	9.9E-1
Diabetes	1.35	1.01–1.81	4.2E-2	1.05	0.75–1.48	7.6E-1	1.35	1.07–1.69	9.9E-3
Cancer	1.22	0.89–1.68	2.2E-1	1.24	1.02–1.50	3.4E-2	0.76	0.59–1.00	4.8E-2
Death	1.32	1.05–1.65	1.7E-2	1.64	1.34–2.00	1.3E-6	1.31	1.01–1.70	4.4E-2

^a^ HRS SNP used is proxy SNP rs222827

N denotes sample size; NA = not available or not estimated

ARIC = the Atherosclerosis Risk in Communities Study, FHS = the Framingham Heart Study (FHS); and HRS = the Health and Retirement Study.

CHD = coronary heart disease; HF = heart failure.

HR denotes hazard ratio from the Cox proportional hazards regression model.

^b^ In HRS HR was evaluated for risk of death. For diseases in HRS we evaluated odds ratios (OR) using logistic regression model because no information on onsets of these diseases was available.

CI denotes confidence interval

The minor allele is also nominally significantly (*p*<5.0×10^−2^) associated with risks of death in two other studies (ARIC and HRS) and with risks of HF (ARIC, FHS), stroke (FHS), diabetes (ARIC, HRS), and cancer (FHS, HRS). It increases risks of all diseases and death (Table 2), except cancer in HRS. All non-significant effects were also detrimental.

### Explicating age-related genetic heterogeneity in the FHS

FHS sample includes two cohorts of participants from different generations (the FHS original cohort and the FHS offspring [FHSO] cohorts) which may be a natural source of age-related genetic heterogeneity (see the Introduction).

Evaluation of risks of the selected diseases and death in each cohort separately (Table 3) shows that weak and highly non-significant association of the rs222826_T allele with risk of diabetes in the entire FHS sample (HR = 1.05, *p* = 7.6×10^−1^; Table 2) is due to antagonistic effects of this allele on risks of diabetes in the FHS original (HR = 0.71, *p* = 2.3×10^−1^) and FHSO (HR = 1.36, *p* = 1.5×10^−1^) cohorts. Formal test shows that multiplicative interaction of the minor allele with these FHS cohorts is significant (HR = 2.5, *p* = 1.1×10^−2^). Explicating this heterogeneity, the effect size in the FHSO (HR = 1.36, *p* = 1.5×10^−1^) became the same as in ARIC (HR = 1.35, *p* = 4.2×10^−2^) and HRS (OR = 1.35, *p* = 9.9×10^−3^). Important result is that we observe the same effect sizes in three cohorts of younger individuals who were born in the same time period around 1930s-1940s (Table 1). The opposite effect is observed in the FHS original cohort for individuals from substantially older generation born around 1910s (Table 1).

**Table 3:** Hazard ratios for major human diseases and death for the rs222826_T (minor) allele carriers in the Framingham cohorts.

Outcome	FHS_C1, N = 1,105			FHSO, N = 3,595		
	HR	95% CI	p-value	HR	95% CI	p-value
CHD	1.20	0.79–1.83	4.0E-1	0.99	0.70–1.41	9.7E-1
CHD_65+_	1.50	0.95–2.37	7.9E-2	NA	NA	NA
HF	1.41	0.87–2.28	1.6E-1	1.70	1.07–2.70	2.5E-2
Stroke	1.91	1.10–3.31	2.1E-2	1.60	0.97–2.65	6.8E-2
Diabetes	0.71	0.40–1.24	2.3E-1	1.36	0.90–2.06	1.5E-1
Cancer	1.21	0.82–1.77	3.4E-1	1.25	1.00–1.58	5.2E-2
ND	1.89	1.24–2.88	2.9E-3	NA	NA	NA
Death	1.45	1.07–1.97	1.7E-2	1.90	1.44–2.50	4.7E-6

N denotes sample size; NA = not available or not estimated

FHS_C1 = the Framingham Heart Study (FHS) original cohort; FHSO = the FHS Offspring cohort.

CHD = coronary heart disease; CHD_65+_ = coronary heart disease with onset at 65 years and older; HF = heart failure; ND = neurodegenerative diseases (dementias including Alzheimer’s type).

HR denotes hazard ratio from the Cox proportional hazards regression model.

CI denotes confidence interval

**Fig 1:**
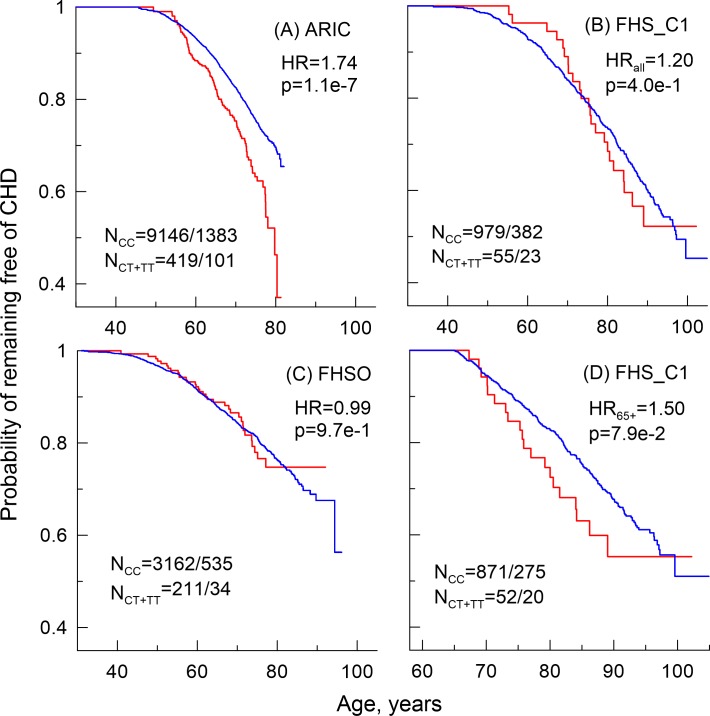
Probability of remaining free of coronary heart disease (CHD) for the rs222826_T allele carriers. (A) The Atherosclerosis Risk in Communities Study (ARIC), (B, D) the Framingham Heart Study (FHS) original (FHS_C1) cohort, (C) the FHS Offspring (FHSO) cohort. HR denotes hazard ratio, HR_all_ in panel (B) denotes the estimates for the entire FHS_C1 sample. HR_65+_ in (D) denotes the estimates for onsets of CHD at ages 65 years and older. N = n/m denotes the size of the entire sample (n) and the number of CHD cases (m) for major allele homozygotes (CC, blue color) and minor allele carriers (CT+TT, red color). P shows p-values.

Kaplan-Meier survival curves (Fig 1A–1C) suggest that in ARIC and each FHS cohort the rs222826_T allele carriers can be at antagonistic risks of CHD at different ages. They can be protected against early onset CHD and be at risk of later onset CHD. These antagonistic effects are most pronounced in the FHS original cohort. This heterogeneity implies that the estimates of the risks in this cohort are biased because of disproportional hazards. Correcting for this heterogeneity by focusing on individuals with onset of CHD at 65 years and older (Fig 1D), the effect size in the FHS original cohort becomes nearly the same as in ARIC and attains suggestive significance, HR = 1.50, *p* = 7.9×10^−2^.

### The rs222826_T allele and risk of neurodegenerative diseases

Given old ages of the genotyped participants of the FHS original cohort, we tested the association of the rs222826_T allele with ND. Table 3 shows that this allele is significantly associated with ND as well.

### Do biomarkers mediate genetic associations with diseases and death?

To address this question, we conducted causal mediation analysis (see Methods). First, we evaluated the associations of the minor allele with the selected biomarkers in each study. The analyses show significant and marginally significant associations of this allele with HDL-C (*β* = -3.8, *p* = 4.7×10^−3^) and TG (*β* = 6.6, *p* = 3.3×10^−3^) in ARIC and BMI (*β* = -3.4, *p* = 5.7×10^−2^) in the FHS original cohort (S1 Table). Then, we evaluated mediating roles of these biomarkers in effects between rs222826 and risks of the selected diseases and death.

The analysis in ARIC (Table 4) showed significant indirect effects of HDL-C and TG in associations of rs222826 with the risks of majority of diseases and death, except cancer (HDL-C and TG) and diabetes (TG). The sizes of these indirect effects were, however, substantially smaller than those of “direct” effects (direct effect means the effect not through the selected mediator). Accordingly, significant mediating effects of lipids explained only a small fraction of the total genetic effects (they are given in Tables 2 and 3).

**Table 4:** Causal mediation analyses: the role of lipids and BMI in the effects of the rs222826_T allele on risks of diseases and death in the ARIC and the FHS original cohort.

Outcome	ARIC, mediator HDL-C				ARIC, mediator TG				FHS_C1, mediator BMI			
	Direct		Indirect		Direct		Indirect		Direct		Indirect	
	HR	p	HR	p	HR	p	HR	p	HR	p	HR	p
CHD^a^	1.66	<8.0E-3	1.05	<8.0E-3	1.66	<8.0E-3	1.04	1.6E-2	1.52	1.0E-1	.989	4.9E-1
HF	1.48	2.4E-2	1.04	<8.0E-3	1.47	2.4E-2	1.05	8.0E-3	1.48	8.8E-2	.958	3.2E-2
Stroke	1.16	6.2E-1	1.03	<8.0E-3	1.13	6.2E-1	1.03	8.0E-3	1.94	5.6E-2	.968	1.9E-1
Diabetes	1.28	1.0E-1	1.07	<8.0E-3	1.33	7.2E-2	1.02	4.0E-1	.752	3.0E-1	.953	4.8E-2
Cancer	1.19	1.7E-1	1.00	4.2E-1	1.18	2.1E-1	1.00	4.3E-1	1.23	3.8E-1	.998	9.5E-1
ND	NA	NA	NA	NA	NA	NA	NA	NA	1.94	<8.0E-3	.958	1.6E-2
Death	1.28	3.2E-2	1.03	<8.0E-3	1.25	5.6E-2	1.03	2.4E-2	1.47	8.0E-3	.981	1.6E-1

HDL-C = high-density lipoprotein cholesterol; TG = triglycerides; CHD = coronary heart disease; HF = heart failure; ND = neurodegenerative diseases (dementias including Alzheimer’s type).

“Direct” (“Indirect”) implies the effect of the rs222826_T allele on an outcome not through (through) a given mediator, i.e., HDL-C or TG in ARIC and BMI in FHS original (FHS_C1) cohort.

HDL-C and BMI were tested for potential mediation effects given their significant associations with the rs222826_T allele in respective studies (S1 Table).

HR denotes hazard ratio from the Cox proportional hazards regression model.

^a^ To correct for disproportionality of hazards, the estimate in the FHS_C1 (original) cohort is given for onset of CHD at ages 65 years and older.

NA = not available

The analyses in the FHS original cohort show significant indirect effects of the rs222826_T allele on risks of HF, diabetes, and ND through BMI (Table 4). As in ARIC, these effects also represented a small fraction of the total effects. However, unlike ARIC, the HRs for the indirect effects were less than one for all diseases. Because the minor allele showed protective effect against diabetes in this cohort (HR = 0.71, *p* = 2.3×10^−1^, Table 3), this indirect effect implied that the association of the minor allele with BMI partly mediated (explained) the association of this allele with diabetes. For HF and ND, conditioning on BMI amplified detrimental effects between the rs222826_T allele and these diseases (compare Tables 3 and 5) because of explicating a fraction of BMI-related genetic heterogeneity.

**Table 5:** Hazard ratios for HF, ND and death for the minor allele of rs222826 or rs222827 SNPs and potential endophenotypes in the FHS original cohort and HRS.

Outcome	Study	Predictor	Sample	HR	95% CI	p
HF	FHS_C1	rs222826_T	All	1.52	0.94–2.46	9.0E-2
		BMI	All	1.05	1.02–1.09	2.0E-3
ND	FHS_C1	rs222826_T	All	2.00	1.31–3.06	1.3E-3
		BMI	All	1.04	1.01–1.08	1.7E-2
Death	HRS	rs222827_A	All	1.32	1.01–1.71	3.9E-2
		Cancer	All	1.29	1.15–1.45	1.4E-5
		rs222827_A	Cancer free	1.51	1.12–2.03	6.7E-3
		rs222827_A	With cancer	0.92	0.53–1.61	7.8E-1

FHS_C1 = the Framingham Heart Study (FHS) original cohort and HRS = the Health and Retirement Study.

HF = heart failure; ND = neurodegenerative diseases (dementias including Alzheimer’s type).

HR denotes hazard ratio from the Cox proportional hazards regression model.

CI denotes confidence interval

All: the models were fitted in the entire samples to estimate additive effects of the minor allele and: (i) BMI measured in kg/m^2^ in FHS and (ii) cancer in HRS.

Rows “Cancer free” and “With cancer” show the results in the HRS samples stratified by cancer status.

### Do diseases mediate genetic associations with death?

Table 6 shows that CHD, HF, and diabetes significantly mediate the risk of death for the rs222826_T allele carriers in ARIC. Diabetes explained 12.5%,—i.e., 4% (HR = 1.04, see Table 6) of 32% (HR = 1.32, see Table 2),—of the death risk. CHD or HF explained 28.1% of the death risk. Mediating effect of either of these diseases (CHD, HF, or diabetes) was also highly significant (HR = 1.11, *p*<8.0×10^−3^) explaining about 34.4% of the death risk.

**Table 6:** Causal mediation analyses: the role of diseases-endophenotypes in the effect of the minor allele of rs222826 ^a^ SNP on risk of death in ARIC, FHS cohorts, and HRS.

Mediator	ARIC				FHS_C1				FHSO				HRS^a^			
	Direct		Indirect		Direct		Indirect		Direct		Indirect		Direct		Indirect	
	HR	p	HR	p	HR	p	HR	p	HR	p	HR	p	HR	p	HR	p
CHD	1.22	1.5E-1	1.09	<8.0E-3	1.42	<8.0E-3	1.00	7.7E-1	1.88	<8.0E-3	1.00	9.4E-1	1.30	7.2E-2	1.01	4.2E-1
CHD_65+_^b^	NA	NA	NA	NA	1.44	4.8E-2	1.00	6.9E-1	NA	NA	NA	NA	NA	NA	NA	NA
HF	1.20	2.0E-1	1.09	2.4E-2	1.43	1.6E-2	1.00	8.9E-1	1.82	<8.0E-3	1.03	1.3E-1	NA	NA	NA	NA
Stroke	1.30	2.4E-2	1.01	5.7E-1	1.39	4.0E-2	1.02	5.6E-2	1.83	<8.0E-3	1.03	2.4E-1	1.31	4.8E-2	1.00	9.2E-1
DM	1.26	1.3E-1	1.04	4.0E-2	1.45	1.6E-2	1.00	8.6E-1	1.84	<8.0E-3	1.02	5.6E-2	1.27	1.1E-1	1.02	8.0E-3
Cancer	1.30	3.2E-2	1.01	1.7E-1	1.41	<8.0E-3	1.00	7.2E-1	1.83	<8.0E-3	1.03	5.2E-1	1.31	2.4E-2	0.99	4.0E-2
ND	NA	NA	NA	NA	1.51	8.0E-3	1.00	8.7E-1	NA	NA	NA	NA	NA	NA	NA	NA
CHD/HF/DM	1.24	1.1E-1	1.11	<8.0E-3	NA	NA	NA	NA	NA	NA	NA	NA	NA	NA	NA	NA

^a^ HRS SNP used is proxy SNP rs222827

CHD = coronary heart disease; CHD_65+_ = coronary heart disease with onset at 65 years and older; HF = heart failure; DM = diabetes mellitus; ND = neurodegenerative diseases (dementias including Alzheimer’s type).

“Direct” (“Indirect”) implies the effect of the rs222826_T allele on risk of death not through (through) a mediator given in column “Mediator.”

HR denotes hazard ratio from the Cox proportional hazards regression model.

CHD/HF/DM denotes the analysis of mediating effect of either of these diseases, i.e., if a person has CHD, or HF, or DM.

^b^ To correct for disproportionality in hazards, the estimate in the FHS_C1 (original) cohort is given for onset of CHD at ages 65 years and older.

NA = not available or not estimated

In FHS (Table 6), only stroke (FHS_C1) and diabetes (FHSO) showed small, marginally significant mediating effects between the rs222826_T allele and risk of death.

In HRS, the minor allele showed small but significant indirect effects on risk of death through diabetes and cancer (Table 6). Indirect effect through diabetes was of mediating nature explaining a small fraction (6.7%) of the total risk of death. Cancer showed moderating effect amplifying the total risk of death for the minor allele carriers in additive and multiplicative approximations (Table 5).

### Pooled effects of the rs222826_T allele on diseases and death and their combined significance

Explicating age-related heterogeneity in the above sections helped in gaining further insights into genetic predisposition to diseases and death and, as a result, in improving estimates of the effects of the minor allele on these outcomes. Accordingly, pooling genetic effects in different populations should leverage these insights.

Table 7 shows the results of meta-analyses leveraging information from the analyses of age-related heterogeneity in FHS (Table 3) and causal inferences (Table 5). These analyses leveraged also potential substantial basis of antagonistic effects for diabetes (see sections “**Explicating age-related genetic heterogeneity in the FHS**” above and “**The role of endophenotypes**” in the Discussion) by pooling evidences for the effects in different FHS cohorts disregarding the effect directions. Meta-analysis of detrimental effects for diabetes, which are seen in younger individuals only, gives HR = 1.35, *p* = 3.3×10^−4^. Further, significant antagonistic effects for cancer in different studies imply significant heterogeneity (e.g., evidenced by non-overlapping 95% CI in Table 2 for cancer estimates in FHS and HRS). Accordingly, the result of meta-analysis in this case was presented in Table 7 disregarding the effect directions. An estimate for cancer in more homogeneous samples of ARIC and FHS is HR = 1.23, *p* = 1.4×10^−2^. For comparison, Table 7 also provides estimates without leveraging all this information.

**Table 7:** The results of meta-analyses of the effects of the minor allele of rs222826 ^a^ SNP on risks of diseases and death.

Outcome	With improvements			No improvements		
	Exp(β)	95% CI	p-value	Exp(β)	95% CI	p-value
CHD	1.35	1.18–1.54	8.9E-06	1.32	1.16–1.50	3.1E-05
HF	1.55	1.24–1.93	9.7E-05	1.51	1.21–1.88	2.4E-04
Stroke	1.25	1.01–1.55	4.0E-02	1.25	1.01–1.55	4.0E-02
Diabetes	1.36	1.16–1.60	1.6E-04	1.22	1.04–1.43	1.5E-02
Cancer	1.25	1.09–1.44	1.8E-03	1.08	0.93–1.25	3.0E-01
ND	2.00	1.31–3.05	1.3E-03	1.89	1.24–2.87	2.9E-3
Death	1.49	1.30–1.70	4.6E-09	1.44	1.27–1.64	3.8E-08
Combined pleiotropic effects p-value			6.6E-21			8.3E-14

^a^ HRS SNP used is proxy SNP rs222827

CHD = coronary heart disease; HF = heart failure; ND = neurodegenerative diseases (dementias including Alzheimer’s type).

Columns “No improvements” (“With improvements”) present the results of meta-analyses without (with) leveraging information from the analyses of age-related heterogeneity and causal inferences.

“Combined pleiotropic effects p-value” is p-value from the Fisher’s combined probability test estimating the global null hypothesis that neither of genetic associations with risks of diseases or death is true.

The sources of information and numerical estimates of the effect estimates used in these meta-analyses are given in S2–S5 Tables.

Exp(β) is exponent of the effect size beta.

CI denotes confidence interval

Table 7 shows pleiotropic associations of the minor allele with risks of all major diseases and death. Combining *p*-values for these pleiotropic associations into a single *p*-value using the Fisher’s test [27], the global null hypothesis that neither of these associations is true is *p* = 6.6×10^−21^. Following meta-analysis without leveraging additional information the estimate is still highly convincing *p* = 8.3×10^−14^. This test provides inflated estimates because it disregards potential correlation of the association signals. However, this is a reasonable approximation for the combined significance of the pleiotropic effects of the minor allele because this *p*-value aggregates the estimates from independent studies.

## Discussion

This paper reports on strong associations of previously non-reported SNPs, rs222826 and its proxy rs222827, located on chromosome 2 in band q22.3, with phenotypes characterizing healthspan (risks of major human diseases including CHD, HF, stroke, diabetes, cancer, and ND) and lifespan (risk of death) in four cohorts from three studies, ARIC, FHS, and HRS. To comprehensively characterize these associations, we adopted an analytic strategy which leveraged the analysis of age-related heterogeneity (this concept is detailed in the Introduction), causal mediation analysis, and information on pleiotropic effects.

### Age-related heterogeneity

Our initial findings (Table 2) show consistent and significant associations of the minor allele (rs222826_T in ARIC and FHS and rs222827_A in HRS) with risks of death in each study but dissimilar associations of this allele with diseases.

Dissimilar associations in different studies may reflect differences in biodemographic structures in these studies [28]. The results of the analyses of age-related heterogeneity in the FHS (Table 3) support this mechanism of non-replication of the genetic associations with risks of diabetes and CHD. Indeed, explicating antagonistic effects between the rs222826_T allele and risk of diabetes in two FHS cohorts shows a striking result that the detrimental effect of this allele is actually the same in three younger cohorts (ARIC, FHSO, and HRS) of individuals born in about the same time period around the 1930s-1940s. The effect is opposite (protective) in substantially older population with mean birth year around the 1910s. For CHD, we observe antagonistic risks at different ages, which cause biased estimates in the models based on the assumption of proportional hazards (Fig 1). Explicating this age-related heterogeneity by focusing on onset of CHD at later ages in the FHS original cohort detected replicating signal. Importantly, the effect size in this case becomes comparable with the effect size in the association of the rs222826_T allele with CHD in ARIC.

Accumulating evidence suggests the importance of age-related heterogeneity in genetic effects. The analyses highlighted the role of age in genetic regulation of BMI [29], sensitivity of the effects of longevity alleles to birth cohorts [30,31], sensitivity of genetic associations with lipids to chronological age [32,33], changes in the allele frequencies with age [34,35], antagonistic risks of diseases and death [16,32].

An important result of these analyses is that they highlight potential non-stochastic mechanisms, which can contribute to non-replication of genetic associations. Clearly, biodemographic factors are not the only ones that can cause non-replication; other factors (e.g., GxG interactions [14]) may play a role.

### The role of endophenotypes

Causal mediation analyses showed that three biomarkers-endophenotypes (HDL-C and TG in ARIC and BMI in the FHS original cohort) significantly moderated the effects between the rs222826_T allele and risks of several diseases and death. They, however, accounted for a small fraction of the genetic effects implying that the major fraction of these effects is not through the selected biomarkers.

As expected, lipids showed significant mediating effects explaining a fraction of the total detrimental effects on cardiovascular diseases in ARIC.

Favorable association of the rs222826_T allele with BMI in the FHS original cohort partly explained its favorable association with risk of diabetes in the same cohort (Table 4). This result emphasizes real nature of the protective (though insignificant) effect of this allele on diabetes. It also shows that favorable associations with diabetes and BMI are characteristic for older people from early birth cohorts (represented by the FHS original cohort). The lack of favorable associations with BMI and the presence of detrimental associations with diabetes in younger cohorts (see the above section) may indicate change in the mechanisms connecting this allele with diabetes in older and younger cohorts [10]. This change is consistent with recent trend on increase of incidence of diabetes [36].

We also found that BMI was a significant moderator in the FHS original cohort amplifying effects between the rs222826_T allele and HF or ND. This moderation effect implies that favorable association of this allele with BMI (S1 Table) can partly mitigate detrimental effects of this allele on HF and ND (compare Tables 3 and 5). Interestingly, this analysis suggests that BMI can be involved in a pathway linking the rs222826_T allele with ND. This is in line with findings in large epidemiological studies reported on association of BMI with ND [37], that is also seen in the FHS original cohort (Table 5).

The mediation analysis of indirect effects of the rs222826_T allele on risks of death through diseases-endophenotypes (Table 6) provided strongest evidence for significant mediating effects of CHD, HF, and diabetes in ARIC. Combined mediating effect of these diseases explained about 34.4% of the death risk. Other diseases in ARIC, FHS, and HRS either mediated substantially smaller fractions of the total effect or did not mediate it at all.

We found that the rs222827_A allele and cancer increase the risk of death in HRS additively (Table 5). However, cancer patients who carry and do not carry this allele show the same survival. This result indicates that detrimental effect of this allele on risk of death can be partly mitigated by (unknown) genetic and/or environmental factors.

Thus, the results from the causal mediation analyses indicate that most of the effects between the minor allele and risks of diseases and death are only partly explained by the selected endophenotypes. These results suggest that such a wide impact of this allele on phenotypes with major contribution to healthspan and lifespan may indicate connections of this variant with some fundamental biological mechanisms (see below) that is in line with the concept of geroscience [21]. Modulation of the effects by age-related heterogeneity and endophenotypes suggests a role of other factors (other genes and/or environment) in the effects of this allele.

### Leveraging analyses of age-related heterogeneity, causal inferences, and pleiotropic effects

A “side effect” of gaining insights into intermediate mechanisms connecting genes with major phenotypes contributing to healthspan and lifespan discussed above is improving statistical estimates (Table 5). Indeed, Table 7 shows improvement in overall significance of the combined pleiotropic effect of the minor allele by seven orders of magnitude from *p* = 8.3×10^−14^ to *p* = 6.6×10^−21^. The minor allele increases risks of death by about 50%; this estimate is genome-wide significant (*p* = 4.6×10^−9^). In addition, the same allele increases risks of CHD by 35% (*p* = 8.9×10^−6^), HF by 55% (*p* = 9.7×10^−5^), stroke by 25% (*p* = 4.0×10^−2^), and ND by 100% (*p* = 1.3×10^−3^). This allele is also associated with risks of diabetes (*p* = 1.6×10^−4^) and cancer (*p* = 1.8×10^−3^). Most of its effects are detrimental as it increases risks of diabetes in younger generations from ARIC, FHSO, and HRS by 35% (HR = 1.35, *p* = 3.3×10^−4^) and risk of cancer in ARIC and FHS by 23% (HR = 1.23, *p* = 1.4×10^−2^).

### Biological role

The rs222826 (and its proxy in HRS, rs222827, which are 90 bp apart) SNP is an intergenic variant with MAF of about 2.5% in each of three Caucasian populations in ARIC, FHS, and HRS. This SNP is located on chromosome 2 in band q22.3, which harbors gene desert region (S1 Fig). Studies show that gene deserts (which make up ~25% of the genome [38]) exhibit characteristics suggestive of functional importance [39]. Functional role of gene deserts is supported by the fact that some of them are evolutionary conserved suggesting their essential role in regulation of core vertebrate genes [40,41].

The rs222826/rs222827 SNPs are within an evolutionary conserved gene desert region [40–42], which contains intergenic regulatory sequences likely involved in regulation of the expression of protein-coding flanking genes *ZEB2* (zinc-finger, E-box-binding homeobox-2) (-1.6 Mb) and *ACVR2A* (activin receptor type-2A) (+1.7 Mb). Function of *ZEB2* can be directed in a tissue- and age-dependent manner by long- (1.2 Mb) and short- (62 Kb) distance enhancers suggesting a conserved regulatory string of enhancers for *ZEB2* and possibly *ACVR2A* [43]. Other long-range enhancers for *ZEB2* were also observed [44,45]. These SNPs are also in LD with SNPs from nearby regulatory regions (e.g., *r*^*2*^ = 1 with rs222809; S2 Fig). In addition, gene expression may be also regulated through non-coding RNAs [46–48].

The *ZEB2* gene functions as a regulator of transcription interacting with activated SMADs in the TGF-β signaling pathway and *ACVR2A* is part of a receptor complex that binds and activates SMAD transcriptional regulators. Accordingly, these genes are linked through SMAD proteins and TGF-β signaling. The *ZEB2* gene is one of key regulators of epithelial-to-mesenchymal transition playing a critical role in the development of neural crest and is involved in the development of other organs that are not derived from the neural crest. This gene mediates multiple pathways related to inflammation, aging and carcinogenesis [49]. The *ACVR2A* gene takes part in many distinct pathways by mediating the functions of members of TGF-β superfamily which are involved in a variety of biological functions including development and tissue homeostasis and associated with a wide range of human diseases [50,51].

Various mutations in *ZEB2* (e.g., haplo-insufficiency, gene inactivation and deletions) and deletions at 2q22-24 are associated with a Mowat–Wilson syndrome, a complex developmental disorder involving a range of physical symptoms as well as severe intellectual disorders [45,52,53]. Detrimental effects caused by the deletions in chromosomal region harboring rs222826/rs222827 and by mutations in flanking genes strengthen functional role of this evolutionary conserved region [44,54].

Potential functional importance of this gene desert is supported by the results of our analyses showing extensive pleiotropic effects on major human diseases and strong effect on human survival.

## Methods

### Data

**The ARIC Study** participants [55] (aged 45–64 at baseline in 1987) were randomly selected and recruited at four field centers across the U.S. We used data from four available examinations. Measurements of biomarkers were available in all examinations. Data on onsets of diseases and survival were available through 2004. Genotyping for 12,771 ARIC participants (N = 9,633 whites) was conducted using Affymetrix 6.0 arrays (1,000K SNPs).

**The FHS** design has been previously described [56–58]. We used data from 28 examinations of the FHS original cohort (aged 28–62 years at baseline in 1948), 8 examinations of the FHS Offspring (FHSO) cohort (aged 5–65 years at baseline in 1970), and one examination of the 3^rd^ Generation (3^rd^ Gen) cohort (aged 21–71 at baseline in 2001). Measurements of biomarkers were available at multiple examinations in the FHS/FHSO and the baseline in the 3^rd^ Gen cohort. Data on onsets of diseases and survival were available through 2011. Biospecimens were mostly collected in the late 1980s and through the 1990s from surviving participants [59,60]. Genotyping of 9,167 white FHS participants was conducted using Affymetrix 500K arrays [58].

**The HRS** design has been previously described [61]. We used available information on biomarkers measured in 2006–2008 and on survival during follow up from 2006 (time of biospecimen collection) through 2013. The data on onsets of diseases was not available. The HRS genotyped about 2.5M SNPs for 12,507 subjects (N = 9,736 whites) using the Illumina HumanOmni 2.5 Quad chip.

### Phenotypes

The focus of the analyses was on risks of major human diseases available in the data including coronary heart disease (CHD), heart failure (HF), stroke, diabetes, cancer, and neurodegenerative diseases (ND, dementias including Alzheimer’s type), and risk of death. Biomarkers represented by the traditional risk factors for cardiovascular diseases were used for causal mediation analyses (see below). They included body mass index (BMI), total cholesterol (TC), high density lipoprotein cholesterol (HDL-C), triglycerides (TG), systolic blood pressure (SBP), and diastolic blood pressure (DBP).

### Analysis

**Fig 2:**
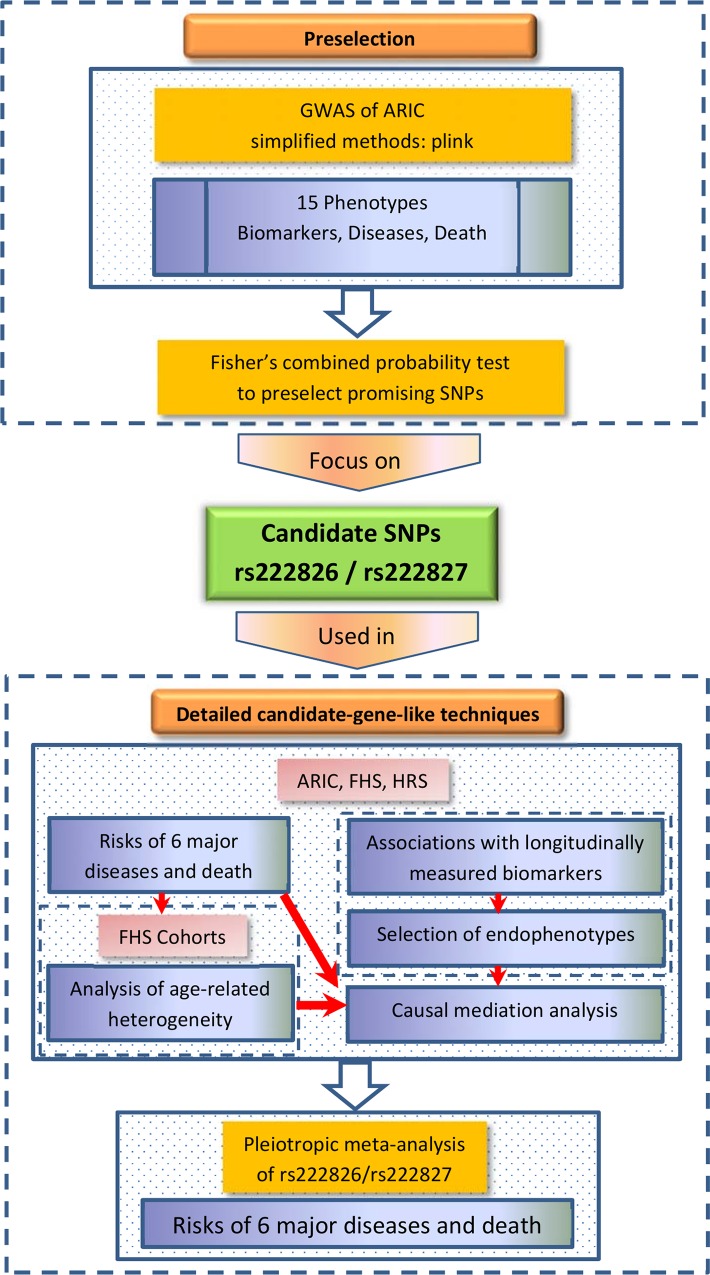
A flowchart of the analyses in this study. All analyses were conducted for whites. Major focus of these analyses was on two uncommon SNPs with minor allele frequency (MAF) ~2.5%. Given this MAF and the available sample size in ARIC, FHS, and HRS, the analyses were conducted for men and women combined to increase the sample of the minor allele carriers.

Because genetic variants may play a complex role in age-related traits (see the Introduction), traditional GWAS techniques, including those designed to evaluate pleiotropic associations [20], may not necessarily address complexity of genetic influence on such traits [28]. Accordingly, the focus of this paper was on comprehensive analyses using more detailed candidate-gene-like techniques. GWAS was used as a tool to preselect variants, which showed promising pleiotropic properties. Below we detail the analyses sketched in the flowchart in Fig 2.

#### Pre-selection

We conducted univariate genome-wide scan using *plink* [62] with the ARIC data set only to preselect SNPs with potential pleiotropic effects. We investigated 15 phenotypes for this scan including BMI, SBP, DBP, TC, HDL-C, TG, ventricular rate, hematocrit, atrial fibrillation, CHD, HF, stroke, cancer, diabetes, and death. Diseases and death were considered as binary outcomes. Linear and logistic regression models were fitted for continuous (baselines measurements were used) and binary outcomes, respectively. These models were adjusted for age, sex, and field center. No other adjustments were used at this stage. Genome-wide scan was conducted using common quality control with the following cut offs: 5% for SNPs and samples missingness and *p* = 10^−4^ for Hardy-Weinberg disequilibrium. MAF filter was >1%. We combined individual *p*-values across all traits using the Fisher’s combined *p*-value [63]. The analyses identified a number of promising SNPs with the Fisher’s *p*-value *p*_*F*_<3×10^−9^ = 5×10^−8^/15.

**Candidate-gene-like analyses** were conducted for the associations of SNPs from a promising pre-selected locus in band 2q22.3 with risks of diseases (see the above section) and death. These analyses were focused on ARIC, FHS, and HRS. The pre-selected SNP rs222826 was directly genotyped in ARIC and FHS. In HRS we used its proxy, rs222827, which was 90 bp away from rs222826. These SNPs were in 100% linkage disequilibrium (LD) in CEU population. These variants were uncommon in Caucasians with MAF of about 2.5% in each of our datasets. We considered a dominant genetic model for the minor allele. Table 1 provides basic characteristics of the selected phenotypes available for the analyses in each study for major allele homozygotes and minor allele carriers.

The data on age at death were available in all studies. The data on onsets of diseases were available in ARIC and FHS. The hazard ratios (HR) of death (all studies) and diseases (FHS and ARIC) were evaluated using the Cox proportional hazards mixed effects regression model (*coxme* package in R) to adjust for potential clustering. Information on both prospective and retrospective onsets of diseases in the FHS was used in these analyses. The use of retrospective onsets in a failure-type model is justified by Prentice and Breslow [64]. These analyses provide estimates of the effects in a given population. The time variable in the Cox regression analyses was the age at onset of an event or at right censoring. In HRS, we evaluated the odds ratios (ORs) for diseases using a logistic regression model (*glm* function in R). Empirical survival age patterns were characterized by the Kaplan-Meier estimator.

**Biomarkers-endophenotypes** for causal mediation analyses were selected based on suggestive and nominally significant (*p*<10^−1^) associations with rs222826/rs222827. These associations were characterized by a linear mixed effects model (*lmer* function in *lme4* package in R). Measurements of BMI, TC, HDL-C, and TG were log-base-10-transformed to offset potential bias due to skewness of their frequency distributions. They were multiplied by 100 for better resolution. Measurements of SBP, and DBP were not transformed as no significant skewness was observed. In the ARIC and FHS datasets, these endophenotypes were measured on multiple occasions during follow-up of the same individuals. We evaluated the associations for SNPs given the measurements of these endophenotypes for individuals of a given age at each examination with available measurements. We used a three-level mixed effects regression model to account for familial and repeated-measurements correlations. Information on longitudinal measurements has multiple advantages including statistical power gain in the analyses [65]. In the HRS dataset, we used single available assessment of these endophenotypes in 2006–2008.

#### Adjustments

All statistical tests were adjusted for: (all studies) age, sex; (ARIC) field center; (FHS) FHS cohorts and whether the DNA samples had been subject to whole-genome amplification [66], and (HRS) HRS cohorts.

#### Causal mediation analysis

We performed a causal mediation analysis to investigate whether any of the effects of rs222826/rs222827 was mediated by endophenotypes. We examined the role of: (i) selected biomarkers as endophenotypes for diseases and death, and (ii) diseases as endophenotypes for death (see section “Phenotypes” above). We followed a unified approach proposed by T. Lange et al. [67] based on marginal structural models (MSMs) [68] to estimate the direct and indirect effects of these SNPs on the hazards. We assumed that there was no confounding between SNP and outcomes due to Mendelian randomization. We included age and sex as covariates in MSMs and assumed that there was no other unmeasured confounders between the endophenotype and outcome conditioned on age and sex. We adopted linear and logistic regression models for the biomarker and disease endophenotypes, respectively, and the Cox regression model for the outcomes. Robust standard errors were obtained using a bootstrap method with 250 replicates to control for the family structure. One of the limitations of the mediation analysis was that it was still possible that there existed other unmeasured confounders between the mediators and the outcomes, and in this case, the mediation effect was not identifiable.

#### Meta-analysis

We adopted a fixed effects model with inverse-variance weighting in the meta-analysis. More specifically, the combined effect size was estimated as $\hat{\beta_{M}}=(\sum_{i}w_{i}\hat{\beta_{i}})/(\sum_{i}w_{i})$ and the variance of this effect size was $var(\hat{\beta_{M})}=1/(\sum_{i}w_{i})$, where $\hat{\beta_{i}}$ is the effect size in the study *i* and *w_i_* is the reciprocal of the variance of $\hat{\beta_{i}}$.

### Accession numbers

This manuscript was prepared using controlled-access data obtained though dbGaP (accession numbers phs000007.v22.p8, phs000280.v2.p1, phs000428.v1.p1). Phenotypic HRS data are available publicly and through restricted access from http://hrsonline.isr.umich.edu/index.php?p=data.

## Supporting Information

S1 Fig Genomic region in band 2q22.3 harboring rs222826 and rs222827 SNPs located 90 bp apart. (PDF)

S2 Fig Linkage disequilibrium (LD) data (*r*^*2*^) for the variant rs222826 in the 1000GENOMES:phase_3:CEU population. Red color denotes regulatory regions and those variants in these regions, which are in LD with rs222826. Inserts show characteristics of SNPs from regulatory regions, which are in LD with rs222826.
(PDF)

S1 Table Associations of the minor allele of rs222826^*^ SNP with biomarkers in ARIC, FHS cohorts, and HRS. (PDF)

S2 Table Sources of information for meta-analysis in Table 7 for columns “With improvements”. (PDF)

S3 Table Sources of information for meta-analysis in Table 7 for columns “No improvements”. (PDF)

S4 Table Numerical estimates of the effect sizes β and standard errors (SE) used for meta-analysis in Table 7 for columns “With improvements”. (PDF)

S5 Table Numerical estimates of the effect sizes β and standard errors (SE) used for meta-analysis in Table 7 for columns “No improvements”. (PDF)
