# Supplementary figures and images for: Pleiotropic Associations of Allelic Variants in a 2q22 Region with Risks of Major Human Diseases and Mortality
Source: PLoS Genet. 2016 Nov 10;12(11):e1006314. doi: 10.1371/journal.pgen.1006314 (PMC5104356; doi:10.1371/journal.pgen.1006314)

**S1 Fig. Genomic region in band 2q22.3 harboring rs222826 and rs222827 SNPs located 90 bp apart.**

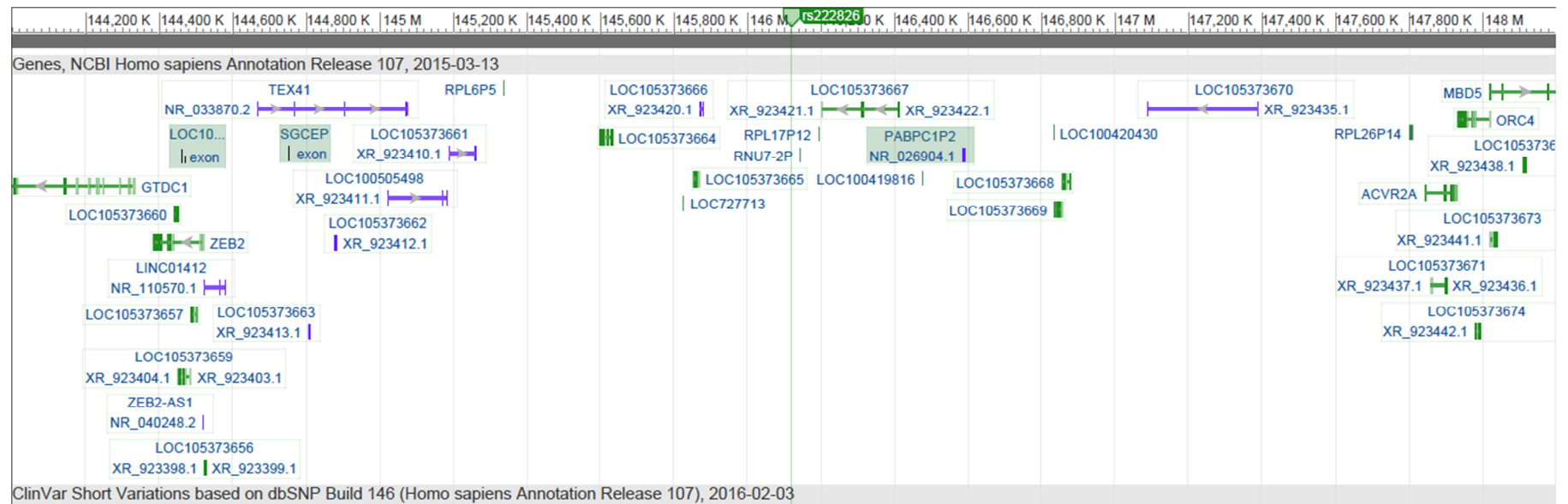

Supplement: S1 Fig — (PDF) [file pgen.1006314.s001.pdf]
