# Supplementary material for: Pleiotropic Associations of Allelic Variants in a 2q22 Region with Risks of Major Human Diseases and Mortality
Source: PLoS Genet. 2016 Nov 10;12(11):e1006314. doi: 10.1371/journal.pgen.1006314 (PMC5104356; doi:10.1371/journal.pgen.1006314)
Supplement: S2 Fig — Red color denotes regulatory regions and those variants in these regions, which are in LD with rs222826. Inserts show characteristics of SNPs from regulatory regions, which are in LD with rs222826. (PDF) [file pgen.1006314.s002.pdf]

S2 Fig. Linkage disequilibrium (LD) data (r^2) for the variant rs222826 in the 1000GENOMES:phase_3:CEU population.

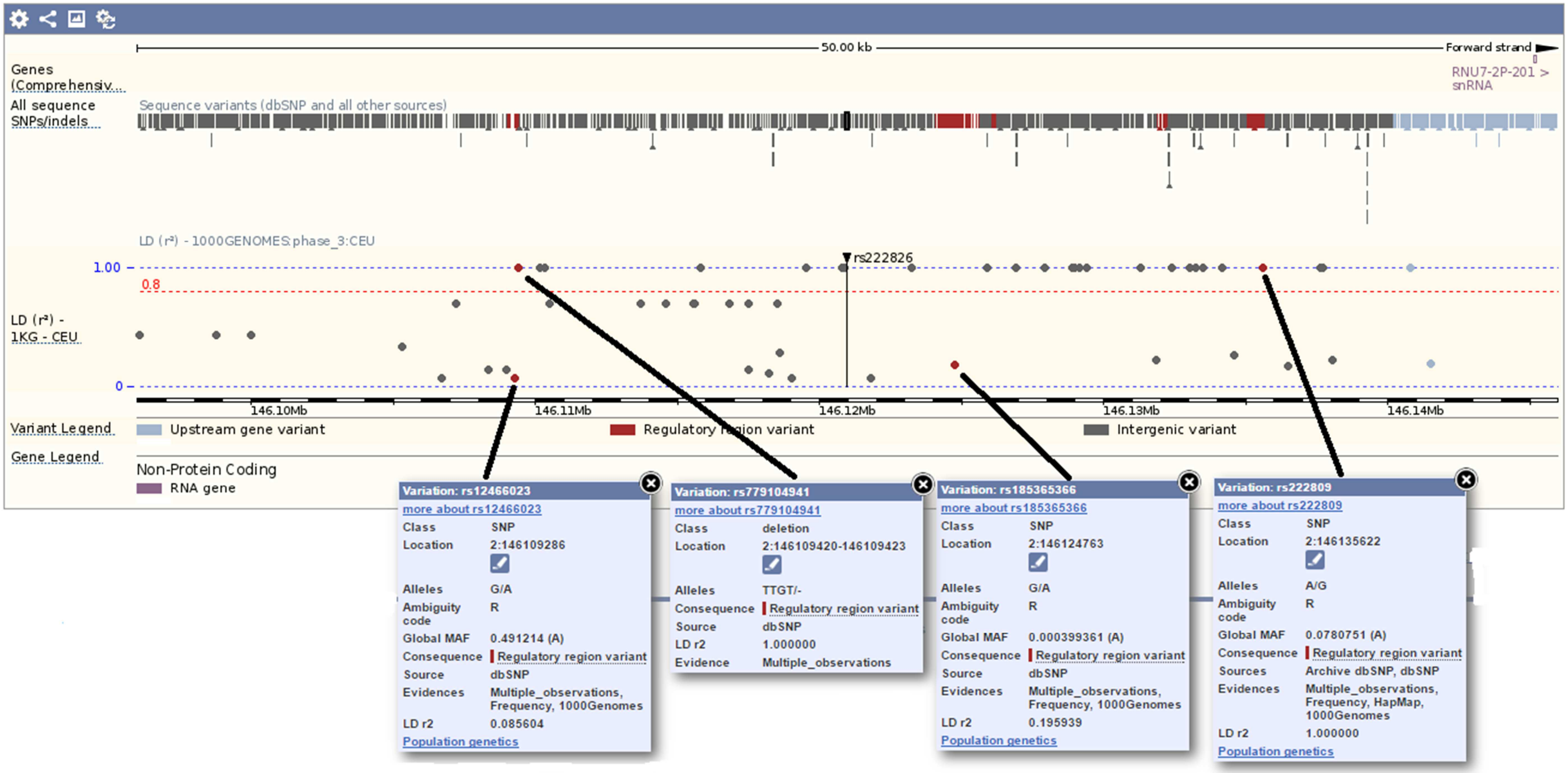

Red color denotes regulatory regions and those variants in these regions, which are in LD with rs222826. Inserts show characteristics of SNPs from regulatory regions, which are in LD with rs222826.
