# Supplementary material for: Pleiotropic Associations of Allelic Variants in a 2q22 Region with Risks of Major Human Diseases and Mortality
Source: PLoS Genet. 2016 Nov 10;12(11):e1006314. doi: 10.1371/journal.pgen.1006314 (PMC5104356; doi:10.1371/journal.pgen.1006314)
Supplement: S1 Table — (PDF) [file pgen.1006314.s003.pdf]

S1 Table. Associations of the minor allele of rs222826[a] SNP with biomarkers in ARIC, FHS cohorts, and HRS.

Outcome	ARIC, N=9,567			FHS_C1, N=1,037			FHSO, N=3,397			HRS			
	β	SE	p	β	SE	p	β	SE	p	N	β	SE	p
BMI	0.706	0.864	4.1E-01	-3.440	1.809	5.7E-02	0.935	1.110	4.0E-01	9,616	0.264	0.922	7.7E-01
TC	0.687	0.808	4.0E-01	2.409	1.919	2.1E-01	0.747	1.016	4.6E-01	7,211	-1.204	1.334	3.7E-01
HDL-C	-3.783	1.338	4.7E-03	2.769	3.262	4.0E-01	-0.085	1.649	9.6E-01	6,097	-1.837	1.748	2.9E-01
TG	6.630	2.255	3.3E-03	-4.253	5.833	4.7E-01	1.702	3.119	5.9E-01	NA	NA	NA	NA
SBP	-0.239	0.780	7.6E-01	-0.493	1.877	7.9E-01	1.284	0.918	1.6E-01	NA	NA	NA	NA
DBP	-0.117	0.417	7.8E-01	-0.023	0.981	9.8E-01	0.122	0.518	8.1E-01	NA	NA	NA	NA

[a] HRS SNP used is proxy SNP rs222827, which is in perfect linkage disequilibrium (LD), r^2=1, with rs222826 in CEU population; these SNPs are 90 bp apart.

N denotes sample size; NA = not available

BMI = body mass index; TC = total cholesterol; HDL-C = high-density lipoprotein cholesterol; TG = triglycerides; SBP = systolic blood pressure; DBP = diastolic blood pressure.

ARIC = the Atherosclerosis Risk in Communities Study, FHS_C1 = the Framingham Heart Study (FHS) original cohort; FHSO = the FHS Offspring cohort, and HRS = the Health and Retirement Study.

The effect size beta is the estimate of cumulative genetic effects over multiple examinations using mixed effects regression model. Sign of beta indicates direction of the effect for the minor allele (dominant genetic model). Values of BMI and lipids (TC, HDL-C, and TG) were log-base-10-transformed to correct for deviations from a normal distribution and multiplied by 100 for better resolution.

SE = standard errors.
