# Supplementary material for: Pleiotropic Associations of Allelic Variants in a 2q22 Region with Risks of Major Human Diseases and Mortality
Source: PLoS Genet. 2016 Nov 10;12(11):e1006314. doi: 10.1371/journal.pgen.1006314 (PMC5104356; doi:10.1371/journal.pgen.1006314)
Supplement: S2 Table — (PDF) [file pgen.1006314.s004.pdf]

Table S2. Sources of information for meta-analysis in Table 7 for columns "With improvements".

Outcomes	ARIC	FHS	FHS_C1	FHSO	HRS
CHD	Table 2		Table 2, CHD_{65+}	Table 3	Table 2
HF	Table 2		Table 5	Table 3	
Stroke	Table 2	Table 2			Table 2
Diabetes	Table 2		Table 3, $\mid \beta \mid$	Table 3	Table 2
Cancer	Table 2	Table 2			Table 2, $\mid \beta \mid$
ND			Table 5		
Death	Table 2	Table 2			Table 5, cancer free

In S2-S5 Tables we used the following notations:
Empty cells = not available or not estimated;
CHD = coronary heart disease;
CHD_{65+} = coronary heart disease with onset at 65 years and older;
HF = heart failure;
ND = neurodegenerative diseases (ND, dementias including Alzheimer's type).
$\mid \beta \mid$ denotes modulus of the effect size beta.
