# Supplementary material for: Pleiotropic Associations of Allelic Variants in a 2q22 Region with Risks of Major Human Diseases and Mortality
Source: PLoS Genet. 2016 Nov 10;12(11):e1006314. doi: 10.1371/journal.pgen.1006314 (PMC5104356; doi:10.1371/journal.pgen.1006314)
Supplement: S3 Table — (PDF) [file pgen.1006314.s005.pdf]

Table S3. Sources of information for meta-analysis in Table 7 for columns "No improvements".

Outcomes	ARIC	FHS	FHS_C1	HRS
CHD	Table 2	Table 2		Table 2
HF	Table 2	Table 2		
Stroke	Table 2	Table 2		Table 2
Diabetes	Table 2	Table 2		Table 2
Cancer	Table 2	Table 2		Table 2
ND			Table 3	
Death	Table 2	Table 2		Table 2

In S2-S5 Tables we used the following notations:
Empty cells = not available or not estimated;
CHD = coronary heart disease;
CHD_{65+} = coronary heart disease with onset at 65 years and older;
HF = heart failure;
ND = neurodegenerative diseases (ND, dementias including Alzheimer's type).
| β | denotes modulus of the effect size beta.
