# Supplementary material for: Pleiotropic Associations of Allelic Variants in a 2q22 Region with Risks of Major Human Diseases and Mortality
Source: PLoS Genet. 2016 Nov 10;12(11):e1006314. doi: 10.1371/journal.pgen.1006314 (PMC5104356; doi:10.1371/journal.pgen.1006314)
Supplement: S4 Table — (PDF) [file pgen.1006314.s006.pdf]

Table S4. Numerical estimates of the effect sizes β and standard errors (SE) used for meta-analysis in Table 7 for columns "With improvements".

	ARIC		FHS		FHS_C1		FHSO		HRS	
	beta	SE	beta	SE	beta	SE	beta	SE	beta	SE
CHD	0.555	0.105			0.408	0.233	-0.006	0.180	0.099	0.113
HF	0.406	0.148			0.417	0.246	0.529	0.236		
Stroke	0.166	0.230	0.530	0.182					-0.003	0.164
Diabetes	0.303	0.149			-0.349	0.289	0.305	0.212	0.298	0.115
Cancer	0.199	0.163	0.212	0.100					-0.269	0.136
ND					0.695	0.216				
Death	0.275	0.116	0.494	0.102					0.412	0.152

In S2-S5 Tables we used the following notations:
Empty cells = not available or not estimated;
CHD = coronary heart disease;
CHD_{65+} = coronary heart disease with onset at 65 years and older;
HF = heart failure;
ND = neurodegenerative diseases (ND, dementias including Alzheimer's type).
| β | denotes modulus of the effect size beta.
